# News and Gossip

**Published:** 1871-03

**Authors:** 


### News and Gossip.

CARL FORMES, the distinguished *basso*, it is said has married a lady physician in Vienna who cured him of an abscess in the throat. Carl is certainly generous. A few years since he presented a Chicago physician with an elegant piano for curing him of a sore throat which impeded his vocal success. So said the physician and the papers.——Twenty-five patients from the Insane Hospital attended the Fechter Matinee, at the Globe Theatre in Boston, recently.——A correspondent sends the *Iowa City Republican*, several columns of which are devoted to a recent resurrection case in that village. Aside from the excitement such affairs usually cause in country towns, there is developed a local professional opposition to the Medical College which is somewhat unexpected. We had supposed that the "Iowa City" school was the especial pet of our bucolic brethren in that State. The relatives and friends of the defunct, together with a number of the local physicians, denounce the Faculty as the authors of the "outrage," but the latter utterly deny the soft impeachment, and claim that they get their *materièl* in States where the laws permit. The *corpus delicti*, duly injected, etc., for use, they claim was procured without their authority or consent. It is a pretty quarrel, as it stands, and we hope the enterprising gentlemen who seek to elevate the profession by starting a new school in a country hamlet, may find their way happily out of it. The fifth wheel never should squeak.——The *Reporter* (Philadelphia) publishes a number of puffs which its subscribers have sent him on renewal of their subscriptions, whereupon the *Richmond and Louisville* editor (with multitudinous associates) strikes out from the shoulder thus: "As to its value [*id est*, 'The largest Medical Journal in America'], there is a heavy [*sic*] file of testimony from distinguished physicians throughout the whole country, but these opinions are never published, because it is justly regarded as coarse, vulgar, and reprehensible to do so." First blood for the Southron.——A reviewer in the *American Journal of Medical Sciences* has a savage critique on Prof. Byford's new book on "The Theory and Practice of Obstetrics." He rages up and down the pages of our venerable, and usually exceedingly staid, contemporary, like a veritable "hot livered grammarian" gone out of his senses. Meanwhile it seems to us funny, and somewhat incomprehensible, that our quarterly *confrere* should have always, heretofore, puffed our respected townsman's books, and at this late day incontinently "goes back on him." The last book is immensely superior, both in doctrine and delivery, to either of its predecessors. "Why is this thus?"——Meanwhile we are reminded of what is said to be a fact by those who were there. A learned teacher (who is said sometimes to be a little careless in remarks he makes to other physicians' patients, as well as in his grammar), on occasion was exhibiting a speculum to the wondering gaze of a dozen or so of medical students, and is credited with saying, "I do not care what may be said of this instrument, I made sixteen thousand dollars last year by it." Happily the speculum furor is abating.——The Homœopathists are irate because one of their fraternity who had been appointed an examining surgeon for pensions, was removed as soon as his *status* became known at headquarters.——Queen

Victoria, like a sensible woman, discountenances the attempt of several of her "strong-minded" to don Æsculapian pantaloons, The short-haired women and long-haired men everywhere lament her position. Why don't they refer it to the "hereditary insanity" of her family?——Porter has been made Admiral (*per aspera*). but he can never be made *admirable* to our professional brethren in the naval service. It appears, however, that the vexed question of the "assimilated rank" is near settlement. The staff officers are to have what the women are after—their rights.——The publishers of a book, noticed in the JOURNAL of last month by our lively correspondent "Volta Reeke," positively adduce, in a public advertisement, the fact of such notice, without indicating its character, as an inducement to the public to purchase their book. We call the attention of the State's Attorney and the Grand Jury to their duty in the premises. The entire edition should be seized and destroyed, and the statute against obscene publications enforced against its getters up. It is time that these filthy emanations from the public press should be stopped. And yet a desperate effort is being made to introduce this worst "Satan in Society" to the center-tables of the very elect.——The Chicago Board of Health, we hear, have at last issued a volume of records of their doings and proposings.——Prof. Skoda, of Vienna, on account of failing health, has resigned the chair of Clinical Medicine.——Brown-Sequard has taken up his residence in Boston, leaving Paris on account of the war.——Helmbold-Buchu entertains the New York Press with splendid dinners, drives an unequaled tandem on Broadway (for which latter he is arrested and fined) and in return is put in the field as a candidate for the presidency by gentlemen whose appetites he has gratified by his viands, and whose smarting urethras, it is fair to suppose, he has appeased by his nostrums.——Dr. Zacheus Bass, upon whom an honorary degree was recently conferred by Rush Med. College, was a surgeon in the war of 1812, and has been in continuous successful private practice ever since. He resides in Middlebury, Vermont.——Efforts are being made to utilize the nutrient material abstracted from meat by the brine used in preserving it. Mr. Whitelaw, of Glasgow, by a process of dialysis, gets a pint of solid extract of meat from two gallons of brine.——Dr. George T. Elliott, Professor of Obstetrics in Bellevue Hospital Medical

College, died Jan. 29th. He had an attack of apoplexy in July last, from which he had apparently recovered. He was widely and favorably known by his contributions to the medical journals, his volume, " Obstetric Clinic, " as well as by his connection with the College.——A monument to " Wm. T. G. S. Morton, Inventor and Revealer of Anæsthetic Inhalation—Before whom in all time Surgery was Agony—By whom pain in Surgery was averted and Annulled—Since whom Science has controlled Pain," has been erected at Mount Auburn, by a committee of the most eminent medical gentlemen in Boston. The committee also propose a national subscription for the family, who are left in straitened circumstances. These things are very "fit to be done." Unlike many epitaphs, this tells the truth fairly, honestly, and yet nobly. We trust the national subscription may prove a great success.——Although chloral in large doses evidently tends to aggravate bronchial congestion, Dr. Caspar Morris commends it in asthmatic bronchitis. He gives five grains (every two hours p. r. n.) in Aubergier's Syrup of Lactucarium.——M. Chaufford (Societé Med. des Hopitaux) uses large doses of carbolic acid in the severe secondary fever of confluent small-pox. He gives one gramme (15.4 grs.) in four or five ounces of menstruum during the day. External lotions of the same are contemporaneously used. He asserts that this treatment causes rapid disappearance of the febrile phenomena of the symptoms of suppuration. Nothing is said with regard to the diet or use of other remedies. A full, nutritious diet with plentiful stimulus, in our experience, is absolutely essential. Possibly the carbolic acid may render other stimulus, save dietetic, unnecessary.——Rumor hath it that Prof. Henry H. Smith, of the University of Pennsylvania, occupant of the chair of surgery since 1855, is about to resign. His successor is not named. Is it "the great Christian Surgeon of the Northwest," (*Vide N. Y. Independent*), the "successor of Dr. Brainard," (*Vide* several other newspapers)? We should be exceedingly loth to lose *him*, but we will send his lithograph in "*The Arts*," with extreme pleasure.——A she-doctor of the infinitessimal persuasion, records a case of tubal pregnancy (*Medical Investigator*) which was cured by *Caul. Puls.* and *Gels.* *Caul.* predominant. Carbolic Acid and Hydrate of Chloral incidentally. *Puls.* was 6th, but *Caul.* was a grain to a goblet of water.

